# Neuroprotective Effects of Cannabidiol but Not Δ^9^-Tetrahydrocannabinol in Rat Hippocampal Slices Exposed to Oxygen-Glucose Deprivation: Studies with *Cannabis* Extracts and Selected Cannabinoids

**DOI:** 10.3390/ijms22189773

**Published:** 2021-09-09

**Authors:** Elisa Landucci, Costanza Mazzantini, Daniele Lana, Pier Luigi Davolio, Maria Grazia Giovannini, Domenico E. Pellegrini-Giampietro

**Affiliations:** 1 Department of Health Sciences, Section of Clinical Pharmacology and Oncology, University of Florence, Viale Pieraccini 6, 50139 Florence, Italy; costanza.mazzantini@unifi.it (C.M.); daniele.lana@unifi.it (D.L.); mariagrazia.giovannini@unifi.it (M.G.G.); domenico.pellegrini@unifi.it (D.E.P.-G.); 2 Farmacia del Madonnone, Via Aretina 9R, 50135 Florence, Italy; farmaciadavolio@libero.it

**Keywords:** Bedrocan extract, FM2 extract, cerebral ischemia, neuroprotection, CB1, CB2, TRPV1, TRPV2, 5-HT1A

## Abstract

(1) Background: Over the past 10 years, a number of scientific studies have demonstrated the therapeutic potential of cannabinoid compounds present in the *Cannabis Sativa* and *Indica* plants. However, their role in mechanisms leading to neurodegeneration following cerebral ischemia is yet unclear. (2) Methods: We investigated the effects of *Cannabis* extracts (Bedrocan, FM2) or selected cannabinoids (Δ^9^-tetrahydrocannabinol (THC), cannabidiol (CBD), and cannabigerol) in rat organotypic hippocampal slices exposed to oxygen-glucose deprivation (OGD), an in vitro model of forebrain global ischemia. Cell death in the CA1 subregion of slices was quantified by propidium iodide fluorescence, and morphological analysis and tissue organization were examined by immunohistochemistry and confocal microscopy. (3) Results: Incubation with the Bedrocan extract or THC exacerbated, whereas incubation with the FM2 extract or cannabidiol attenuated CA1 injury induced by OGD. Δ^9^-THC toxicity was prevented by CB1 receptor antagonists, the neuroprotective effect of cannabidiol was blocked by TRPV2, 5-HT1A, and PPARγ antagonists. Confocal microscopy confirmed that CBD, but not THC, had a significant protective effect toward neuronal damage and tissue disorganization caused by OGD in organotypic hippocampal slices. (4) Conclusions: Our results suggest that cannabinoids play different roles in the mechanisms of post-ischemic neuronal death. In particular, appropriate concentrations of CBD or CBD/THC ratios may represent a valid therapeutic intervention in the treatment of post-ischemic neuronal death.

## 1. Introduction

*Cannabis* (*Cannabis Sativa*), also known as marijuana, has been used as a medicinal and recreational drug for centuries. It contains more than 120 different active principles whose effects are not fully understood yet [1]. The two most rigorously investigated phytocannabinoids are Δ^9^-tetrahydrocannabinol (THC) and cannabidiol (CBD). THC accounts for most of the psychotropic effects of *Cannabis* (including perception alterations, rewarding effects, hyperphagia, and abuse potential), while CBD appears to lack these typical “Δ^9^-THC-like” properties [2]. The role of *Cannabis* in medicine is rapidly evolving due to the novel advancements in the understanding of the pharmacology of *Cannabis* that have led to numerous proposed clinical uses. Nowadays, in many countries, including Italy, it is possible to administer *Cannabis* extracts or drugs for therapeutic purposes under medical prescription for some specific uses, including chronic pain in multiple sclerosis, nausea and vomiting caused by chemotherapy, anorexia, and cachexia in cancer patients or patients suffering from AIDS, and for the reduction in involuntary body and facial movements in the Gilles de la Tourette syndrome [3,4].

Cerebrovascular diseases are a major cause of disability worldwide in the aged population [5]. Forebrain global ischemia occurs as a result of an abrupt reduction in cerebral blood flow following cardiac arrest, cardiomyopathies, or aortic rupture [6] and leads to a decrease in oxygen and nutrient supply to the brain, causing delayed neuronal loss and neurodegeneration, particularly in the CA1 area of the hippocampus. The role of cannabinoids in cerebral ischemia is extremely controversial [7]: whereas CB1 agonists have shown to be protective in ischemic in vivo and in vitro models [8,9], other studies have shown a “dark side of cannabinoids” in which agonists are toxic or antagonists are protective [10,11,12]. Interestingly, accumulation of anandamide results in neurotoxicity, whereas accumulation of 2-arachidonoylglycerol results in neuroprotection [13,14,15].

As for the natural *Cannabis* active principles, recent studies have shown that CBD reduced the infarct volume when administered ≤ 6 h after stroke onset [16]. Repeated treatment with CBD improves functional outcome and survival rates, which suggests that CBD may have neuroprotective effects at both the early phase and late time point. In mouse and piglet models of stroke, CBD significantly increases cerebral blood flow [17]. Moreover, CBD reduces brain edema and blood-brain barrier permeability associated with ischemic conditions [18]. In a clinical trial, a single intravenous dose of dexanabinol (HU-211), a synthetic and nonpsychoactive cannabinoid derivate, in patients experiencing severe traumatic brain injury is not efficacious, but it is safe [19].

However, some concern exists regarding the use of marijuana and the occurrence of acute ischemia. The increased incidence of cerebral ischemia in young adults may be in part due to improved stroke detection, greater prevalence of known stroke risk factors, and more recreational and illicit drug use [20]. Recent systemic review evidenced an association between exposure to *Cannabis*-based products and cardiovascular disease. Currently, this evidence is stronger for ischemic stroke than for any other cardiovascular disease [21]. Prevalence of heart failure, cerebrovascular accident (CVA), coronary artery disease, sudden cardiac death, and hypertension was significantly higher in patients with *Cannabis* use [22]. For example, a study from the U.S. nationwide inpatient sample showed that recreational use of *Cannabis* is independently associated with a 2.25-fold increase in the risk of acute ischemic stroke among people aged 25 to 34 years [23].

In a previous study, we have demonstrated that activation of CB1 receptors by exogenous CBs consistently exacerbated post-ischemic damage and that, whereas the endocannabinoid 2-AG produced neuroprotection, anandamide caused toxicity [13]. In this study, we examined the effects of natural *Cannabis* extracts or selected *Cannabis* active principles in rat organotypic hippocampal slices exposed to OGD, an in vitro model of cerebral ischemia. To explore the putative underlying mechanisms, we also investigated the dependence of these effects on CB1, CB2, TRPV1, TRPV2, 5- HT1A, or PPARγ receptors.

## 2. Results

### 2.1. Effects of Cannabis Extracts and Selected Cannabinoids against OGD Neurotoxicity in Organotypic Hippocampal Slices

**Figure 1:**
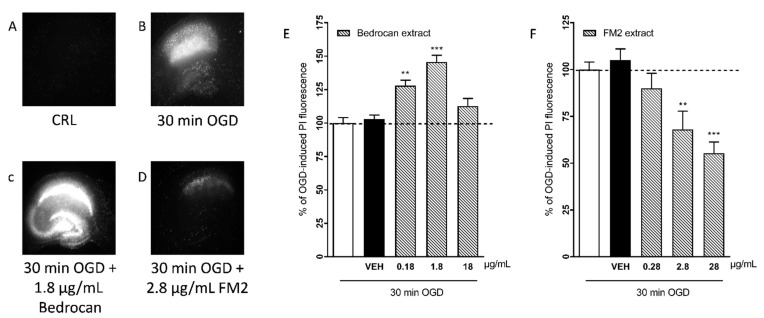
Qualitative and quantitative analysis of the effects of Bedrocan and FM2 extracts on OGD toxicity in rat organotypic hippocampal slices. OGD was applied for 30 min, and 24 h later, the damage in the CA1 region was assessed, measuring the intensity of PI fluorescence. Drugs were present in the incubation medium during OGD and the subsequent 24 h recovery period. (**A**) Hippocampal slice under normal conditions (background PI fluorescence), slice exposed to 30 min OGD displaying intense PI labeling in the CA1 subregion (**B**), CA1 damage induced by 30 min OGD was exacerbated by the presence of 1.8 g/mL of Bedrocan extract (**C**) CA1 damage induced by 30 min OGD was attenuated by the presence of 2.8 g/mL of FM2 extract (**D**). (**E**) The Bedrocan extract at the doses of 0.18 and 1.8 mg/mL significantly exacerbated CA1 injury in comparison to the damage caused by 30 min OGD alone, considered as 100%. (**F**) The FM2 extract dose-dependently attenuated CA1 injury. Data are expressed as percentage of OGD-induced PI fluorescence. Bars represent the mean ± SEM of at least 5 experiments run in quadruplicate. (** *p* < 0.01 and *** *p* < 0.001 vs. OGD; one-way ANOVA plus Dunnett’s test).

Our HPLC titration revealed that the Bedrocan extract contained 16.69 mg/mL of Δ^9^-THC and 0.66 mg/mL of CBD; the contents of Δ^9^-THC and CBD in the FM2 extract were 4.6 mg/mL of Δ^9^-THC and 8.2 mg/mL of CBD as reported in Table 1 and traces of delta -9-tetrahydrocannabinolic acid (THC-A), cannabidiolic acid (CBD-A) and cannabinol (CBN). Rat organotypic hippocampal slices incubated with increasing concentrations of the Bedrocan and FM2 extract formulations displayed no damage in the CA1 region, as evaluated using PI fluorescence (data not shown). When cultures were subjected to 30 min OGD, we observed a selective injury in the CA1 area [13]. When present in the incubation medium during OGD and the subsequent 24 h recovery period, the Bedrocan extract (0.18–1.8 µg/mL) corresponding to 0.1–10 µM of THC concentration produced a significant exacerbation of the CA1 injury that was 145% of the OGD control at 1.81 µg/mL (Figure 1E). In contrast, under similar conditions, the FM2 extract (0.28–28 µg/mL) corresponding to 0.1–10 µM of CBD concentration dose-dependently attenuated CA1 injury, reaching a maximal reduction of 45% at 28 µg/mL (Figure 1F).

**Figure 2:**
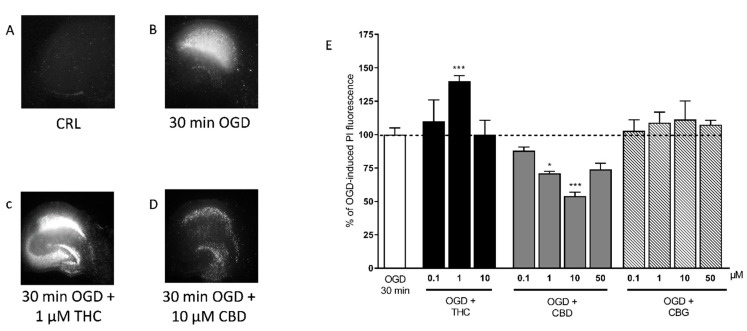
Qualitative and quantitative analysis of the effects of selected cannabinoids in rat organotypic hippocampal slices exposed to OGD. (**A**) Hippocampal slice under normal conditions (background PI fluorescence), slice exposed to 30 min OGD displaying intense PI labeling in the CA1 subregion (**B**), CA1 damage induced by 30 min OGD was exacerbated by the presence of 1 µM THC (**C**) CA1 damage induced by 30 min OGD was attenuated by the presence of 10 µM CBD (**D**). (**E**) THC, the psychoactive constituent of *Cannabis*, significantly exacerbated the neurotoxic effects induced by OGD at the concentration of 1 µM. CBD significantly attenuated CA1 injury with the maximal effect at 10 µM, and CBG had no effect. Bars represent the mean ± SEM of at least 5 experiments run in quadruplicate. * *p* < 0.05 and *** *p* < 0.001 vs. OGD (one-way ANOVA plus Dunnett’s test).

Because Bedrocan and FM2 extracts contain a mixture of cannabinoids, we analyzed the effects of selected natural cannabinoids, such as THC, CBD, and also CBG. When THC was present in the incubation medium during the 30 min of OGD and the subsequent recovery period, we observed a significant exacerbation of CA1 injury at 1 µM (Figure 2), which was similar to what was seen with the Bedrocan extract. Conversely, CBD significantly attenuated the CA1 injury at the concentrations of 1 and 10 µM (Figure 2), similar to what was observed with the FM2 extract. CBG had no effects at all doses tested (0.1–50 µM).

**Figure 3:**
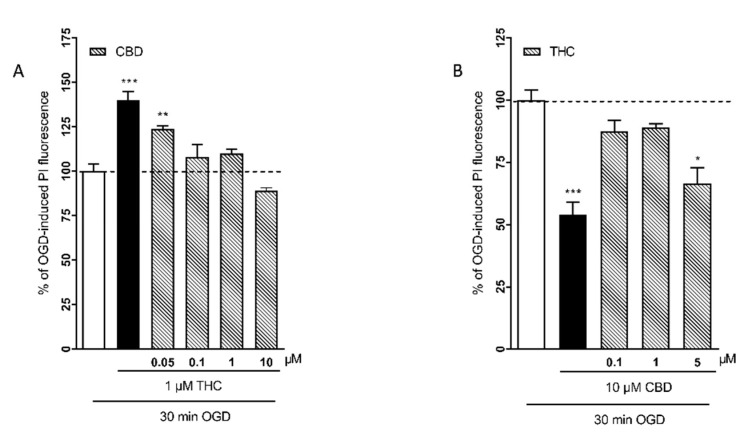
Effects of different combinations of THC and CBD concentrations in rat organotypic hippocampal slices exposed to OGD. (**A**) When the slices were incubated with the relative concentrations of CBD increased (0.1–10 µM), the neurotoxic effect of THC was lost. (**B**) The combination of 10 µM CBD with 5 µM THC was neuroprotective, but when the relative concentrations of THC were lower, the effect was lost. Bars represent the mean ± SEM of at least 5 experiments run in quadruplicate. * *p* < 0.05, ** *p* < 0.01 and *** *p* < 0.01 vs. OGD (one-way ANOVA plus Dunnett’s test).

In order to reproduce the variability of diverse cannabinoid concentrations in the different *Cannabis* flos products, we examined the effects of different combinations of THC and CBD concentrations. Figure 3A shows the results of incubating the slices with 1 μM THC alone or in combination with increasing concentrations (0.05–10 μM) of CBD. Figure 3B shows the results of the incubation with 10 µM CBD in combination with increasing concentrations (0.1–5 µM) of THC. The combination of 1 µM THC with 0.05 µM CBD, which reproduces the cannabinoid concentrations of Bedrocan 1.8 µg/mL, produced a significant neurotoxic effect, but when the relative concentrations of CBD increased (0.1–10 µM), the neurotoxic effect was lost (Figure 3A). The combination of 10 µM CBD with 5 µM THC, which reproduces the cannabinoid concentrations of FM2 28 µg/mL, was neuroprotective, but when the relative concentrations of THC were lower, the effect was lost (Figure 3B).

**Figure 4:**
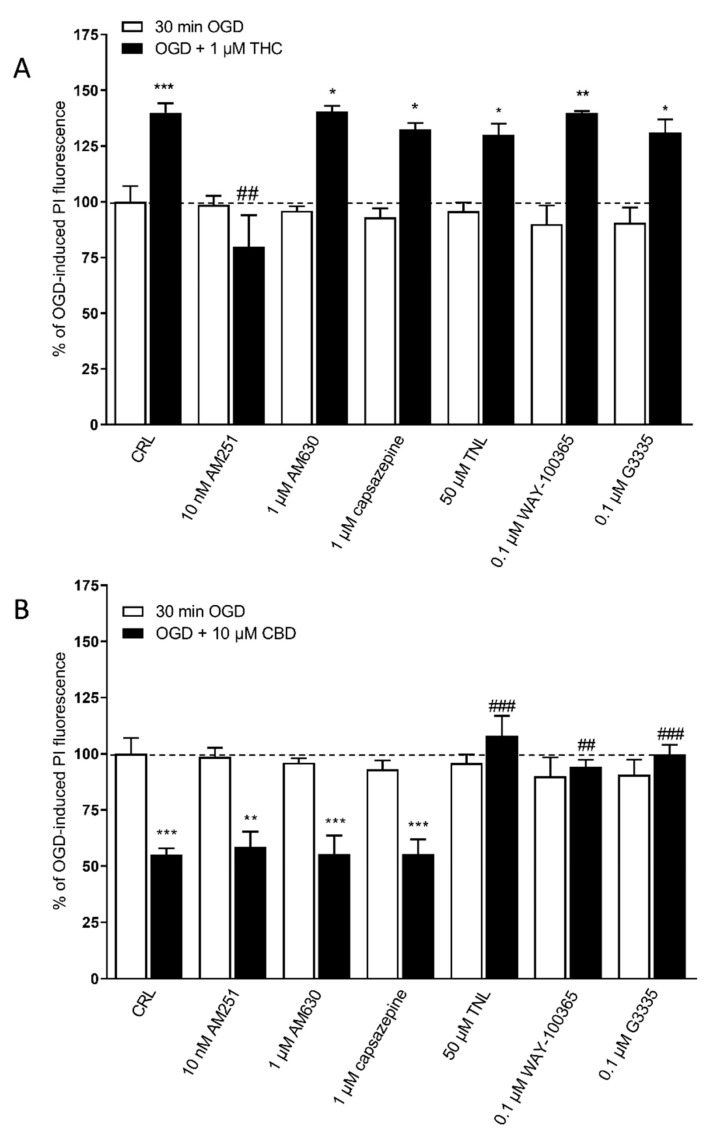
The effects of THC and CBD on OGD toxicity in rat organotypic hippocampal slices depend on different receptors. (**A**) The neurotoxic effects of 1 µM THC on CA1 were completely prevented only by co-incubation with the CB1 receptor antagonists AM251. (**B**) On the other hand, the neuroprotective effect of 10 µM of CBD was completely prevented by co-incubation with the TRPV2 antagonist tranilast (TNL), or 5HTA1 antagonist WAY-100635, or PPARγ antagonist G3335, but not by the CB1 receptor antagonists AM251, the CB2 antagonist AM630, and the TRPV1 antagonist capsazepine. Bars represent the mean ± SEM of at least 5 experiments run in quadruplicate. * *p* < 0.05, ** *p* < 0.01 and *** *p* < 0.001 vs. OGD; ## < 0.01vs. OGD + THC for (A); ## < 0.01vs. OGD + CBD and ### < 0.001vs. OGD + CBD for (B) (one-way ANOVA plus Tukey’s *w-*test).

In a previous paper, we had observed that the neuroprotective effects of 2-AG were dependent on CB1 receptors and the neurotoxic effects of AEA on CB1 and TRPV1 receptors [13]. Similary, to understand the possible mechanism of action underlying THC toxicity and/or CBD protection in our model, we examined the effects of specific antagonists of CB1, CB2, TRPV1, TRPV2, 5-HT1A, and PPARγ receptors, at concentrations that displayed no effect when used alone (Figure 4). We observed that the exacerbation of OGD toxicity caused by 1 µM THC was significantly attenuated only by the CB1 receptor antagonist AM251 at 10 nM, but not by any of the other antagonists that were investigated (Figure 4A). On the other hand, the neuroprotective effect of 10 µM CBD was significantly prevented by the TRPV2 antagonist tranilast (50 µM), the 5-HT1A antagonist WAY-100365 (0.1 µM) and the PPARγ receptor antagonist G3335 (0.1 µM) but not by the CB1 antagonist AM251(10 nM), the CB2 antagonist AM630 (1 µM) and the TRPV1 receptors antagonist capsazepine (1 µM) (Figure 4B).

### 2.2. CBD Rescues Neuronal Damage in OGD-Treated Slices

We assessed the effects of CBD and THC on CA1 pyramidal cell viability using immunostaining for NeuN on organotypic slices harvested 24 h after 30 min OGD followed by immunofluorescence and confocal microscopy.

**Figure 5:**
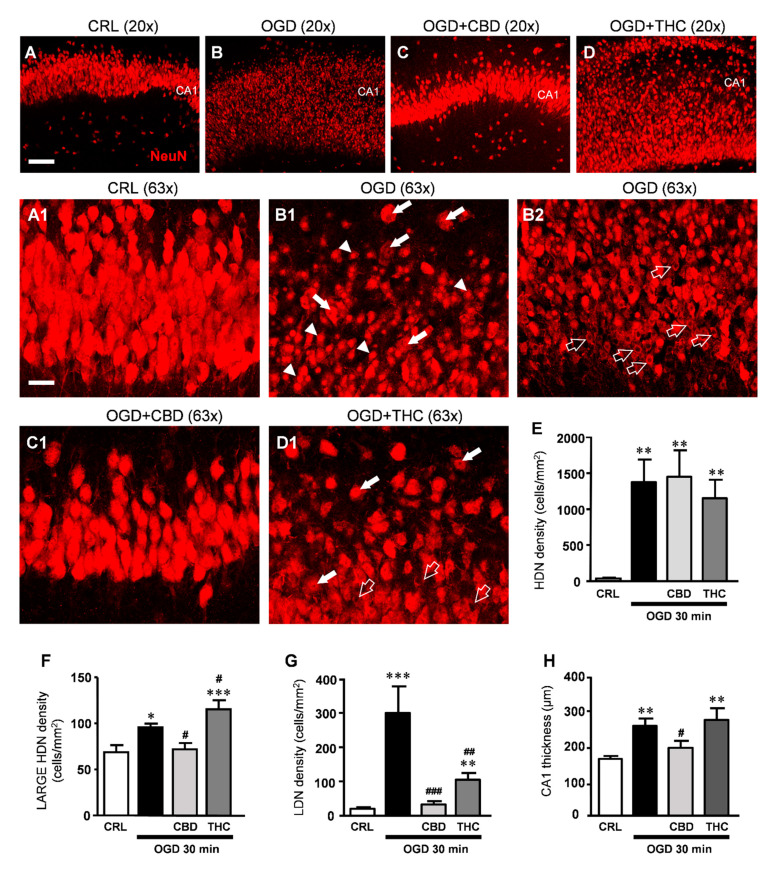
(**A**–**D**) Representative confocal images of neurons immunostained using antibodies against NeuN in CA1 of a CRL (**A**), OGD (**B**), OGD + CBD (**C**), and OGD + THC slice (**D**) captured with a 20x objective, scale bar: 75 µm. (**A1**–**D1**) Confocal images obtained stacking 10 consecutive z-scans acquired in the depth of the corresponding slice shown in (**A**–**D**), respectively (0.3 µm each, total thickness 3 µm), captured with a 63x objective. A1: Image taken from the control slice in A showing healthy pyramidal neurons in CA1. (**B1**,**B2**) Both images of CA1 were taken from the OGD slice in (**B**) and show clearly that neurons are profoundly damaged after OGD. (**B1**) HDN neurons (arrowheads), large HDN neurons (white arrows); (**B2**) LDN neurons (open arrows). (**C1**) The image of CA1 taken from the OGD + CBD slice in (**C**) shows that pyramidal neurons have a healthy appearance. (**D1**) The image taken from the OGD + THC slice in (**D**) shows large HDN neurons (white arrows) and LDN neurons (open arrows). (**A1**–**D1**) scale bar: 20 µm. (**E**–**G**) Quantitative analyses of neuronal morphological alterations in CA1. (**E**) density of HDN neurons. Statistical analysis: one-way ANOVA *p* < 0.001; ** *p* < 0.01 vs. CRL, Newman–Keuls post hoc test; (**F**) density of large HDN neurons. Statistical analysis: One-way ANOVA *p* < 0.0001; * *p* < 0.05 and *** *p* < 0.001 vs. CRL, # *p* < 0.05 vs. OGD, Newman–Keuls post hoc test. (**G**) density of LDN neurons. Statistical analysis: One-way ANOVA *p* < 0.0001; ** *p* < 0.01 and *** *p* < 0.001 vs. CRL, ### *p* < 0.001 vs. OGD, ## *p* < 0.01 vs. OGD and OGD + CBD, Newman–Keuls post hoc test). (**H**) Thickness of CA1. Statistical analysis: One-way ANOVA *p* < 0.01; ** *p* < 0.01 vs. CRL, # *p* < 0.05 vs. OGD, Newman–Keuls post hoc test. Bars represent the mean ± SEM of at least 8 experiments.

The qualitative analysis of NeuN-positive neurons in organotypic hippocampal slices (Figure 5B,B1,B2) shows that OGD caused complex morphological modifications in many neurons of the CA1 stratum pyramidalis similar to what described in a previous study [24]. As shown in panels B1-B2, a significantly high number of neurons had a highly condensed, pyknotic nucleus (high-density nucleus, HDN, neurons, arrowheads in B1) that were distributed along the entire thickness of the CA1 region. Many neurons showed an enlarged, vacuolized cytoplasm and high-density nuclei (large HDN neurons, white arrows in B1), other neurons showed signs of karyorrhexis, demonstrated by the lack of their nuclear staining (low-density nucleus, LDN, neurons open arrows in B2). Therefore, NeuN immunostaining confirmed that viability of CA1 pyramidal neurons exposed to 30 min OGD was significantly decreased in comparison to control slices.

The quantitative analyses were performed counting HDN, large HDN, and LDN neurons separately, which were all increased following OGD (Figure 5E–G). Treatment with CBD or THC did not prevent the development of HDN neurons in the CA1 stratum pyramidalis caused by OGD (Figure 5E). Conversely, CBD significantly prevented the increased density of large HDN induced by OGD, while THC increased its density in comparison to OGD (Figure 5F). Furthermore, the dramatic increase in the density of LDN neurons induced by OGD was reverted to almost control levels by CBD; the incubation with THC also prevented the effect of OGD, but to a lesser extent as compared with CBD (Figure 5G). Finally, it is evident from the representative figures shown in panels B, B1, and B2 compared to panels A and A1 that OGD caused not only a modification of neuronal morphology but also disorganization of the tissue. Indeed, we found that the thickness of CA1 was increased by about 59% following OGD. CBD significantly reverted this disorganization of the tissue (Figure 5C1,H) while THC had no effect (Figure 5D1,H). All these data, taken together, demonstrate that CBD had a significant protective effect against CA1 neuronal damage and tissue disorganization caused by OGD in organotypic hippocampal slices. The protective effect of THC was less consistent.

## 3. Discussion

In this study, we show that the Bedrocan extract or THC exacerbated whereas the FM2 extract or cannabidiol attenuated CA1 injury induced by OGD. Δ^9^-THC toxicity was prevented by CB1 receptor antagonists, the neuroprotective effect of cannabidiol was blocked by TRPV2, 5-HT1A, and PPARγ antagonists. Confocal microscopy confirmed that CBD, but not THC, had a significant protective effect toward neuronal damage and tissue disorganization caused by OGD in organotypic hippocampal slices.

Medical *Cannabis* and individual cannabinoids, such as Δ^9^-tetrahydrocannabinol (THC) and cannabidiol (CBD), are receiving growing attention from both the media and the scientific literature. In the present study, we examined for the first time the effects of both *Cannabis* extracts and selected cannabinoids in an in vitro model of brain ischemia routinely used in our laboratory, using rat organotypic hippocampal slices exposed to OGD [13,25,26]. *Cannabis* is the most commonly produced and consumed illicit substance in the world, and the contents of THC and CBD vary widely in “street” *Cannabis*. A recent study quantified the potency of THC and CBD content in *Cannabis* seized by police in England in 2005 and found that the median content of THC was significantly higher than the one recorded 10 years earlier [27]. In contrast, in a meta-analysis [28] performed to assess the potency of THC from 1970 to 2009, CBD content was found to be extremely low in more recent *Cannabis*. These findings indicate that the preference for *Cannabis* variants containing higher THC content carries significant health risks, particularly to those who are susceptible to its harmful effects. Therefore, the growing popularity of recreational consumption of *Cannabis*, especially among the young population, raises immediate concerns about its safety. Indeed, recent case-control studies and systemic reviews have shown that *Cannabis* use can significantly affect physical and mental health, lead to substance dependence, and induce adverse neurovascular effects such as ischemia in the young population [21,29].

The role of cannabinoids in cerebral ischemia is extremely controversial [7]. In our previous study, we observed that activation of CB1 receptors by exogenous CBs consistently exacerbates post-ischemic damage, whereas endocannabinoids have a different outcome, depending on the molecule used. In particular, 2-AG had neuroprotective effects, while AEA resulted in neurotoxic [13]. A recent systemic review and meta-analysis reported that all subclasses of cannabinoids, Cannabis-derived, synthetic, specific CB1R, and CB2R agonists, significantly reduce infarct volume in transient and permanent ischemia and improve both early and late functional outcomes in experimental stroke when given after stroke onset [16].

In our experiments, we observed that the Bedrocan extract and THC caused neurotoxicity, shown by an increase in PI fluorescence in slices exposed to 30 min OGD, whereas the FM2 extract resulted neuroprotective. These different and opposite effects of the two *Cannabis* extracts could depend on the different concentrations of the most abundant cannabinoids contained in the plant. In addition, large concentration variabilities among different extracts from the same starting typology of *Cannabis* are often found [30].

It has been shown that CBD has a trend to reduce the infarct size with delayed administration ≤ 6 h after stroke onset [16]. Repeated treatment with CBD improves functional outcome and survival rates, suggesting that CBD may have neuroprotective effects not only at the early phase but also at the late time point after infarct [31]. Multiple targets have been proposed to mediate the neuroprotective effects of CBD, such as a combination of a potent antioxidant, immunosuppression, and anti-inflammatory actions [32]. CBD also counteracts cerebral hemodynamic impairment and produces beneficial cardiac effects. A systemic review and meta-analysis with 25 studies has concluded that CBD is associated with changes in hemodynamics in vivo. Acute and chronic administration of CBD do not affect blood pressure or heart rate under control conditions but reduce blood pressure and heart rate in stressful conditions [33]. In mouse and piglet models of stroke, CBD significantly increases cerebral blood flow [17,34]. Moreover, CBD reduces brain edema and blood-brain barrier permeability associated with ischemic conditions [18]. Our results demonstrate that the benefits of the neuroprotection of FM2 extract (rich in CBD) and CBD may also originate from a direct impact on the neuroprotection via reducing cell death/disruption.

Previous preclinical studies have demonstrated that cannabidiol (CBD) elicits neuroprotective effects in models of neurodegeneration [35] and oxidative stress [36], but it becomes neurotoxic at higher concentrations. Similarly, we observe in this study that CBD at higher concentrations loses its neuroprotective effects, although it does not induce neurotoxicity in our model. Similarly, THC produced a bell-shaped dose-response curve for its neurotoxic effects, as we [13] and others [37] have observed repeatedly with endogenous and exogenous cannabinoids in in vitro studies.

Previous preclinical studies have demonstrated that cannabidiol (CBD) and cannabigerol (CBG), two non-psychotomimetic phytocannabinoids from *Cannabis Sativa*, induce neuroprotective effects on neurodegeneration [27]. In the same study, high concentrations of CBD were toxic since a 40–50% reduction in cell viability was observed [27], in accordance with our experiments in which CBD at a high dose lost its neuroprotective effects.

A number of studies have shown that CBG has neuroprotective potential in vitro and in animal models to reduce the severity of neurologic illnesses, such as Huntington disease (HD), amyotrophic lateral sclerosis, Parkinson disease, and multiple sclerosis; these effects seem to be mediated largely through PPARγ receptors [38]. Nevertheless, CBG has no effects in our “ex vivo model”.

The pharmacology of ∆^9^-THC is relatively well-established: THC is a CB1R and CB2R partial agonist [39]. The pharmacology of CBD is less clear. CBD may operate through components of the endocannabinoid system with low affinity for CB1 and CB2 receptors [39] or on different neurotransmitter systems and pathways. Other CBD effects may be mediated by direct activation of 5-hydroxytrypamine 1A (5-HT1A) receptors [34,35,40,41,42,43,44,45,46] and peroxisome proliferator-activated receptorγ (PPARγ) [17,34]. In addition, CBD is shown to have an antagonistic effect against THC [35,40]. Interestingly, among all the above-highlighted mechanisms, the 5-HT1A receptor seems to be the most strongly implicated in the regulatory effects of CBD on mood and mental diseases [40]. Several pharmacological approaches with selective receptor antagonists such as WAY-100635 showed that anxiolytic-, antidepressant-, and antipsychotic-like effects of CBD are predominantly mediated by 5-HT1A-Rs [41,42,43]. 5-HT1A is a crucial serotonergic receptor subtype involved in many functions of serotonin in the brain, and several works have associated many beneficial actions of CBD to this receptor, in accordance with our results [34,44,45]. In vitro study reported by Russo and collaborators suggests that CBD could facilitate 5-HT1A-mediated neurotransmission by acting as an agonist at this receptor [45]. However, more recent findings indicate that CBD is not a full 5-HT1A receptor agonist as was originally proposed, but its mechanism of action seems to involve allosteric interactions with the receptor or interference with the intracellular pathways associated with this receptor [46]. In an in vivo model of transient global ischemia, Mori and colleagues investigated the ability of CBD to prevented anxiety-like behavior, memory impairments, and despair-like behaviors; in particular, the anxiolytic-like effects of CBD in global ischemia were attenuated by CB1, CB2, 5-HT1A, and PPARγ receptor agonists [47]. Different studies suggested that hippocampal neuronal-synaptic modulation by TRPV1 is a possible mechanism of action for CBD [48,49,50]. CBD has been suggested to be a potent activator of TRPV2 [51,52]. Recently cryo-electron microscopy (cryo-EM) was used to elucidate the structure of TRPV2 in a CBD-bound state [53]. Iannotti et al. demonstrated that besides TRPV1, CBD activates and rapidly desensitize other TRP channel including TRPV2 [54]. In our study, we show that the exacerbation of OGD toxicity caused by THC was significantly attenuated only by the CB1 receptor antagonist AM251 but not by any of the other antagonists that were investigated. On the other hand, the neuroprotective effect of 10 µM CBD was significantly prevented by the 5-HT1A antagonist WAY-100365 and the PPARγ receptor antagonist G3335 and for the first time in this model by the TRPV2 antagonist tranilast, but not by the CB1 antagonist AM251, the CB2 antagonist AM630 and the TRPV1 receptors antagonist capsazepine.

In previous papers from our [24,55] and other labs [56], different forms of damaged neurons were identified and quantified in in vitro and in vivo models of ischemia. Neurons with marked signs of pyknosis defined as high-density nuclei (HDN) and karyorrhexis defined low-density nuclei (LDN) neurons are found in CA1 after an ischemia-like insult [24]. In the present model, we found a third example of neuronal degeneration, large high-density nuclei (large HDN) neurons. In CA1 of OGD slices, we found a significantly higher number of all these forms of damaged neurons (HDN, large HDN, and LDN neurons) in comparison to control slices. The interesting data is that when OGD slices were treated with CBD, we found clear signs of neuroprotection. In particular, we found a significant decrease in large HDN neurons and of LDN neurons in comparison with OGD slices. THC did not show neuroprotective effects similar to those of CBD.

The measure of the width of the CA1 layer further characterized the ischemia-induced alteration of the tissue. In OGD-treated slices, CA1 appeared abnormally and significantly thicker than in control slices, and the tissue was disorganized and disomogeneous, with loss of the interneuronal contacts in the pyramidal layer. Treatment with CBD but not THC prevented these ischemia-dependent effects. The disorganization of the tissue could be associated with an OGD-induced loss of integrity of the extracellular matrix (ECM) that constitutes and guarantees the scaffolding of the neuron-neuron and neuron-glia interactions. Indeed, the ischemic insult initiates a series of cytoplasmatic and nuclear events that degrade cytoskeletal proteins, such as actin and spectrin [57], as well as extracellular matrix proteins, such as laminin [58]. ECM changes in composition and assembly in neurological disorders compromise the physiological neurons-glia, neuron-neuron interactions [59]. This pathological disassembly of the ECM had also been demonstrated in different experimental models of ischemia [60,61]. It will be of extreme interest to understand through which mechanism CBD allows the maintenance of tissue integrity after the ischemic insult.

## 4. Materials and Methods

### 4.1. Animals

Male and female Wistar rat pups (7–9 days old) were obtained from Charles River (Milano, Italy). Animals were housed at 23 ± 1 °C under a 12 h light-dark cycle (lights on at 07:00) and were fed a standard laboratory diet with ad libitum access to water. Experiments and animal use procedures were carried out in accordance with the National Institutes of Health Guide for the Care and Use of Laboratory Animals (NIH Publications No. 80–23, revised 1996). The authors further attest that all efforts were made to minimize the number of animals used and their suffering.

### 4.2. Materials

AM251, AM630, capsazepine, cannabigerol (CBG), and cannabidiol (CBD) were purchased from Tocris Cookson (Bristol, UK). Tissue culture reagents were obtained from Gibco-BRL (San Giuliano Milanese, MI, Italy) and Sigma (St. Louis, MO, USA). Δ^9^-tetrahydrocannabinol (THC), tranilast, and WAY-100635 were purchased from Sigma (St. Louis, MO, USA). G3335 was purchased from Cayman (Ann Arbor, MI, USA). *Cannabis* flos forms were Bedrocan^®^ (Bedrocan BV, Veendam, The Netherlands), with reported Δ^9^-THC and CBD concentrations of 22% and <1%, respectively, and FM2^®^, available only within the Italian territory and produced by the Military Pharmaceutical Institute of Florence, with reported Δ^9^-THC and CBD concentrations of 5–8% and 7.5–12%, respectively.

### 4.3. Preparation of Extracts from Bedrocan and FM2

In accordance with the Legislative Decree 09/11/2015 of the Italian Ministry of Health, which indicates to prepare the *Cannabis* extracts according to the Good Preparation Rules FU. Because the decarboxylation reaction temperature for cannabinoids is around 110 °C [62], Bedrocan or FM2 *Cannabis* flos samples were heated at 115 °C for 70 min in a high-precision forced natural convection stove. Subsequently, *Cannabis* samples were heated in a water bath at 100 °C for 40 min together with appropriate solvents (Bedrocan 100 mg/mL in alcohol at 96° Vol and propylene glycol 50/50 *v/v* and FM2 70 mg/mL in olive oil) according to the method “SF” of the Società Italiana Farmacisti Preparatori (SIFAP) [63]. The mixture of solvents and *Cannabis* were and then filtered. In order to prevent oxidation, 10 mg/mL of natural vitamin E FU and 0.2 mg/mL of BHT-butylhydroxytoluene were added to the FM2/olive oil extract.

To titrate the extracts, we used a Jasco HPLC system UV-970 (Thermo Fisher Scientific, Waltham, MA, USA). Chromatographic separations were achieved using an Eppendorf CH-30 analytical column (Eppendorf, Sigma, St. Louis, MO, USA), the detection wave-length was 228 nm, and the injection volume 50 µL. The working conditions were as follows: the mobile phase consisted of a mixture of ACN/water in 75/25 *v/v* ratio, the flow rate was 0.3 mL/min, and the column temperature was 40 °C.

The quantitative analyses were performed by the external standard of 5 pure samples (CBN, CBD, Δ9-THC, CBD-A, and THC-A) with a known title of cannabinoids 1 mg/mL.

### 4.4. Oxygen and Glucose Deprivation (OGD) in Rat Organotypic Hippocampal Slices

Organotypic hippocampal slice cultures were prepared as previously reported [20]. Briefly, hippocampi were removed from the brains of male and female 7–9-days-old Wistar rats (Charles River, MI, Italy), and transverse slices (420 μm) were prepared using a McIlwain tissue chopper and transferred onto semiporous membranes inserts and maintained in culture for 14 days in vitro. The slices were subjected to OGD by exposure to a serum-free medium devoid of glucose and previously saturated with 95% N2/5% CO2. Following 30 min incubation at 37 °C in the airtight anoxic chamber, the cultures were transferred to an oxygenated serum-free medium containing 5 mg/mL glucose and returned to the incubator under normoxic conditions until the neuronal injury was evaluated 24 h later [21,64]. The drugs were incubated during the 30 min of OGD and the subsequent 24 h recovery period. Cell injury was assessed in organotypic hippocampal cultures using propidium iodide (PI), a polar dye that enters the cells only if the membrane is damaged and becomes fluorescent upon binding to DNA. PI was added to the medium at the end of the 24 h. Thirty minutes later, fluorescence was viewed using an inverted fluorescence microscope (Olympus IX-50; Solent Scientific, Segensworth, UK) equipped with a xenon-arc lamp, a low-power objective (4X), and a rhodamine filter. Images were digitized using a video image obtained by a CCD camera (Diagnostic Instruments Inc., Sterling Heights, MI, USA) controlled by software (InCyt Im1TM; Intracellular Imaging Inc., Cincinnati, OH, USA) and subsequently analyzed using the Image-Pro Plus morphometric analysis software (Media Cybernetics, Silver Spring, MD, USA). In order to quantify cell death, the CA1 hippocampal subfields were identified for OGD toxicity was identified and encompassed in a frame using the drawing function in the image software (ImageJ; NIH, Bethesda, Rockville, MD, USA), and the optical density of PI fluorescence was detected. THC was dissolved in methanol and stored at −20 °C. WAY-100635 was dissolved in water. CBD, CBG, AM251, AM630, TNL, and G3335 were dissolved in dimethyl sulfoxide (DMSO) and stored at −20 °C. Prior to the application of the drugs to hippocampal slices cultures, they were diluted in cell culture medium. Each drug was added to slices with a maximal final solvent concentration of 0.1% (*v*/*v*) DMSO or 0.3% (*v*/*v*) methanol in cell culture medium. Control experiments with equimolar concentrations of DMSO or ethanol alone did not show any significant effects on the parameters investigated in this study (data not shown).

### 4.5. Immunohistochemistry

Immunohistochemistry was performed with the free-floating method as previously described [65]. Briefly:

Day 1: Organotypic hippocampal slices were placed in a multiwell and washed 3 times for 5 min in PBS-TX (Triton X-100, Sigma-Aldrich, Burlington, MA, United States) then blocked for 60 min with blocking buffer (BB) containing 10% normal goat serum (Vectashield, Burlingame, CA, USA). The slices were then incubated overnight at 4 °C under slight agitation with a mouse anti-NeuN to immunostain neurons (1:400 in BB; Millipore, Billerica, MA, USA).

Day 2: After three washings in PBS-TX, the slices were incubated for 2 h at room temperature in the dark with AlexaFluor555 donkey anti-mouse IgG (1:400 in BB; Thermo Fisher Scientific, Waltham, MA, USA). After three washings in PBS-TX, the slices were mounted onto gelatin-coated slides using Vectashield mounting medium with DAPI (Vectashield, Burlingame, CA, USA).

### 4.6. Microscopy Techniques, Qualitative and Quantitative Analysis

Confocal microscopy acquisitions were performed in the regions of interest (stratum pyramidalis and stratum radiatum) of the CA1 dorsal hippocampus to acquire immunofluorescence signals of neurons. Slices were observed under a LEICA TCS SP5 confocal laser scanning microscope (Leica Microsystems, Wetzlar, Germany) equipped with 20× and 63× objectives. The parameters of acquisition were maintained as follows: frame dimension 1024 × 1024 pixels, frequency of acquisition 200 Hz, z step 1.2 μm with the 20× objective; frame dimension 1024 × 1024 pixels, frequency of acquisition 100 Hz, z step 0.3 μm with the 63× objective

Quantitative analysis of neuron density and CA1 thickness were performed on stacks of 10 consecutive confocal z-scans (1.2 µm each, total thickness 12 µm, acquired with the 20× objective) using ImageJ software (National Institute of Health, http://rsb.info.nih.gov/ij (accessed on October 2020 to June 2021)). Qualitative analyses on neuron morphology were performed on confocal images obtained stacking 10 consecutive z-scans acquired in the depth of the slice (0.3 µm each, total thickness 3 µm, 63× objective). Areas of region of interest in CA1 were calculated in mm^2^, and the density of immunopositive HDN (high-density nucleus) neurons, large HDN neurons, LDN (low-density nucleus) neurons were expressed as cells/mm^2^. HDN neurons are neurons with pyknotic, condensed NeuN-positive nuclei and very faint NeuN-positive cytoplasmic labeling [24]. Large HDN neurons are morphologically larger than HDN neurons and have very faint NeuN-positive cytoplasmatic labeling, while the nucleus shows a highly condensed NeuN and an enlarged, vacuolized cytoplasm. LDN neurons show karyorrhexis, which represents an index of damaged nuclei, while NeuN-positive immunofluorescence persists in the cytoplasm [24]. To characterize the disorganization of the CA1 pyramidal cell layer, we evaluated the thickness of the CA1 stratum pyramidalis: in each NeuN z-projection image, 3 measurements evenly distributed throughout SP were taken and averaged.

### 4.7. Statistical Analysis

Data are presented as means ± SEM of n experiments. The statistical significance of differences between PI fluorescence intensities was analyzed using one-way ANOVA with a post hoc Dunnett and Tukey’s *w*-test for multiple comparisons. Immunohistochemistry data were statistically analyzed by one-way analysis of variance (ANOVA) followed by Newman–Keuls multiple comparison test. All statistical calculations were performed using GRAPHPAD PRISM v. 8 for Windows (GraphPad Software, San Diego, CA, USA). A probability value (*p*) of <0.05 was considered significant.

## 5. Conclusions

In conclusion, our results show that cannabinoids, investigated in an in vitro model of forebrain global ischemia, exert neurotoxic or neuroprotective effects depending not only on the cannabinoid used and on their respective ratios in both natural extracts and laboratory combinations but also on their activity on receptors of different systems. In particular, the Bedrocan extract and THC were neurotoxic, whereas the FM2 extract and CBD were neuroprotective. As also confirmed by the confocal microscopy immunocytochemistry data. The effect of THC appeared to be dependent on CB1 receptors. The effect of CBD was blocked by TRPV2, 5HT1-A, and PPARγ receptor antagonists, but not by antagonists of CB receptors. Combinations of THC and CBD reproducing that existing in *Cannabis* extracts confirmed their toxic or protective effects, but when the ratios were altered, the effects were lost. Therefore, appropriate concentrations of CBD or CBD/THC ratios can be considered as a promising therapeutic strategy in the treatment of post-ischemic neuronal death.

## Figures and Tables

**Table 1:** Cannabinoid compounds identified in Bedrocan and FM2 extracts.

	Concentration (mg/mL)	
Compounds	Bedrocan	FM2
CBD-A	0.39	0.2
CBD	0.66	8.2
CBN	0.32	0.26
Δ-9-THC	16.96	4.6
THC-A	0.61	0.9

## Data Availability

Our own data presented in this study are available on request from the corresponding author.
